# Pimecrolimus 1% cream for anogenital lichen sclerosus in childhood

**DOI:** 10.1186/1471-5945-4-14

**Published:** 2004-10-14

**Authors:** Stefanie Boms, Thilo Gambichler, Marcus Freitag, Peter Altmeyer, Alexander Kreuter

**Affiliations:** 1 Department of Dermatology and Allergology, Ruhr-University Bochum, Gudrunstr. 56, D-44791 Bochum, Germany; 2 Department of Dermatology, Oldchurch Hospital, Waterloo Road, Romford, RM7 OBE, London, United Kingdom

## Abstract

**Background:**

Lichen sclerosus is a chronic inflammatory disease with a predilection of the anogenital region. Because of the potential side effects of repeated local application of potent glucocorticosteroids, equally-effective, safer therapeutic options are required, especially in the treatment of children.

**Case presentations:**

We report on the efficacy of twice-daily application of pimecrolimus 1% cream in four prepubertal girls (range of age: 4 to 9 years) who suffered from anogenital lichen sclerosus. After three to four-month treatment, all patients had almost complete clinical remission including relief from itch, pain and inflammation. Only minor improvement was observed for the white sclerotic lesions. No significant side effects have been observed.

**Conclusions:**

Topical pimecrolimus appears to be an effective and safe treatment for children with anogenital lichen sclerosus. The clinical benefits observed in the four patient presented particularly include relief of pruritus, pain and inflammation. Vehicle-controlled studies on a larger number of patients are now warranted to substantiate our promising findings, and to investigate long-term efficacy and safety of topical pimecrolimus in anogenital lichen sclerosus.

## Background

Lichen sclerosus (LS) is an inflammatory sclerotic skin disease of unknown origin. LS affects all age groups and occurs in about 15% in female children mostly involving the anogenital region [1]. Major subjective complains are severe pruritus, dysuria, painful defecation and vaginism. Clinically, LS is characterized by porcelain-white sclerotic plaques [1]. Histological features include orthokeratotic hyperkeratosis, vacuolar degeneration of the basal layer, oedematous and sclerotic papillary dermis as well as lymphohistiocytic infiltrates in the mid-dermis [2,3]. The disease runs a relapsing course indicating an ongoing inflammatory process. Although the exact pathogenesis of LS is still unclear, the recognized active involvement of skin immune system (e.g., activated T cells and CD1a+/HLA-DR+ dendritic cells) and the association with autoimmune disease and human leukocyte antigen DQ7 in women and girls with LS suggests an immunogenetic component to the disease [2,4]. Topical glucocorticosteroids (GCS) are the first-line therapeutic option for genital LS. Oestrogen or testosterone containing ointments are usually of limited efficacy. Surgery including cryotherapy and laser treatment should be reserved for patients with adhesions or symptomatic patients who fail to respond to multiple medical treatments, since there is a high recurrence rate following surgery [1,5]. Because of the chronic course of genital LS and the potential side effects associated with potent topical GCS or aggressive surgical treatments, alternative well-tolerated therapies are required, especially in the treatment of childhood LS. We therefore investigated the efficacy and safety of topical pimecrolimus in anogenital LS in four prepubertal girls.

## Case presentations

We describe four prepubertal girls between the age of 4 and 9 years who suffered from LS of the anogenital region (Tab. 1). Because of the unequivocal clinical appearance and the patients' age, we renounced a biopsy proven diagnosis. All patients had extensive pruritus, two of them (patient no. 1 and 3) showed additional burning pain, dysuria and increased vulnerability with bleeding. Patient 1 suffered also from painful defecation. Three patients (patient no. 1, 2 and 4) showed partly deep fissuring in the affected areas. All patients had characteristic whitish, sclerotic skin changes in the vulvar and perianal region, three had additional involvement of the perineal region (patient no. 1, 3 and 4). Previous therapies of the patients included emollients and topical antifungal agents (patient 2 and 4). Six months before pimecrolimus treatment patient no. 4 had underwent cryotherapy and steroid instillation with a relapse after six weeks (Table 1).

**Table 1:** Clinical data of patients treated with pimecrolimus 1% cream twice daily

Patient (No)/age (years)	Duration of disease (years)	Previous therapies	Clinical features pre-therapy	Involvement	Duration of therapy (weeks)	Clinical features post-therapy
1/5	2.5	emollients	a, b, c, d, e, f, g	genital, perineal, perianal	12	(f)
2/4	0.3	nystatin cream	a, f, g	genital, perianal	12	f
3/9	0.3	polidocanol cream	a, f	genital, perineal, perianal	12	(f)
4/6	2.5	nystatin cream/cryotherapy/steroid instillation	a, b, c, e, f, g	genital, perineal, perianal	16	f

a = pruritus; b = burning pain; c = dysuria; d = painful defecation; e = bleeding; f = whitish sclerotic lesions: g, = fissuring

Pimecrolimus 1% cream (Elidel^®^, Novartis Pharma, Basel, Switzerland) was applied twice daily in a thin layer to the affected areas. Clinical examination and recording of patients symptoms was performed before, after six weeks, and after three and four months of therapy, respectively. On the six-week follow-up visit, substantial improvement of pruritus, dysuria, and painful defecation has been reported by the patients which had already occurred during the first weeks of treatment. Accordingly skin lesions had improved as well. The patients were then encouraged to continue the treatment twice daily for further six and eight weeks, respectively (Table 1). At the end of therapy, almost complete remission of symptoms was achieved in all four patients, except for the white sclerotic skin changes that showed only minor improvement (patient no. 1 and 3). Side effects observed included transitory mild burning at the initiation of treatment. Two patients (no. 2 and 4) showed no recurrence of disease activity three months after discontinuation of therapy, two patients (no. 1 and 3) were lost on post-treatment follow-up.

## Conclusions

Pimecrolimus belongs to the ascomycin class of macrolactam immunosuppressives, acting by the inhibition of T-cell activation via the calcineurin pathway and inhibition of the release of numerous inflammatory cytokines, thereby preventing the cascade of immune and inflammatory signals. In contrast to GCS, there is no potential to induce skin atrophy [6]. Pimecrolimus 1% cream has been approved for the treatment of atopic dermatitis. It has been proven to be effective in various inflammatory skin diseases, e.g., cutaneous lupus erythematosus, vitiligo and psoriasis [7]. In large studies it has been demonstrated that treatment is well tolerated in paediatric patients and even infants with atopic dermatitis. Three weeks of therapy regardless of the skin areas treated resulted in low blood concentrations without any accumulation at which no systemic effect is expected [6,8]. We therefore refrained from blood monitoring.

Successful treatment of genital LS with calcineurin inhibitors has been reported recently. Böhm et al. [9] used topical tacrolimus 0.1% ointment in three prepubertal girls and three adults with anogenital LS. A complete remission was obtained in all patients and therapy was well tolerated. Additionally, two other case reports of genital LS who responded to tacrolimus ointment have been published [10,11]. Recently Goldstein et al. [12] reported for the first time a 10-year old girl with genital LS who was successfully treated with pimecrolimus ointment. The efficacy of calcineurin inhibitors in LS are mainly due to their immunosuppressive and anti-inflammatory effects. Moreover recent studies indicate a release of neuropeptides from sensory nerve fibres during tacrolimus and pimecrolimus treatment [13]. Accordingly we observed that subjective symptoms such as pruritus and pain completely resolved after a few weeks of treatment and clinical features such as fissuring, purpura, inflammatory erythema and genital bleeding almost completely resolved at the end of therapy (Table 1). The white sclerotic lesions could however not be changed significantly.

On the basis of our case observations, pimecrolimus ointment seems to be effective in LS. However, we cannot fully exclude that the benefit observed was due to emollient effect of the ointment. For example, it has recently been demonstrated in a vehicle-controlled study that the beneficial effect of testosterone ointment claimed in the past was probably due to the emollient effect only [14]. Nevertheless all of our patients had quite severe disease and were previously treated with different emollients without significant success. Hence pimecrolimus was very likely the active part of the treatment. Furthermore in view of the natural course of LS and the severity of patients' disease spontaneous resolution can widely be excluded.

In conclusion, pimecrolimus 1% seems to be a safe and effective treatment modality in pre-pubertal children with anogenital LS. Our case observations provide further evidence for the beneficial effects of topical pimecrolimus on pruritus and inflammatory features. As the recurrence rate of active LS in prepuberty is relatively high and many of the patients have continuing symptoms after menarche as well, a long-term treatment regime not based on GCS is needed to avoid the well-known side effects linked to GCS. Vehicle-controlled studies on a larger number of patients are now warranted to substantiate our promising findings, and to investigate long-term efficacy and safety of topical pimecrolimus in anogenital LS.

## List of abbreviations

Lichen sclerosus: LS; glucocorticosteroids: GCS

## Competing interests

The authors declare that they have no competing interests.

## Authors' contributions

SB conceived of this case study including its conduction and drafting of the manuscript. TG and AK participated in the literature search and critically revised the manuscript. All authors read and approved the final manuscript.

**Figure 1:**
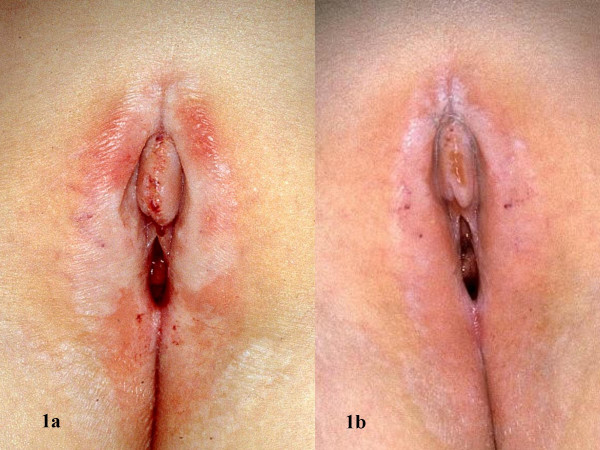
Vulvar region before (a) and after (b) 12 weeks of pimecrolimus 1% cream twice daily (Patient no. 1)

**Figure 2:**
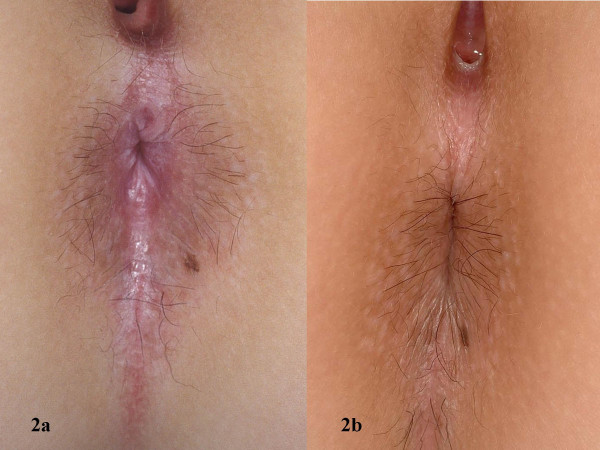
Perianal region before (a) and after (b) 12 weeks of pimecrolimus 1% cream twice daily (Patient no. 3)

## Pre-publication history

The pre-publication history for this paper can be accessed here:


